# Translation, Adaptation and Validation of the Greek Version of the Kerlan-Jobe Orthopaedic Clinic Shoulder and Elbow Score in Greek Overhead Athletes

**DOI:** 10.3390/jfmk9010049

**Published:** 2024-03-12

**Authors:** Eleftherios Paraskevopoulos, Anna Christakou, Stefanos Karanasios, Amalia Panagiota Louka, George M. Pamboris, Maria Papandreou

**Affiliations:** 1 Biomechanics Laboratory, Department of Physiotherapy, University of Peloponnese, 23100 Sparta, Greece; a.christakou@go.uop.gr; 2 Laboratory of Advanced Physiotherapy, Department of Physiotherapy, University of West Attica, 12243 Athens, Greece; skaranasios@uniwa.gr (S.K.); amlouk9@gmail.com (A.P.L.); mpapand@uniwa.gr (M.P.); 3 Department of Health Sciences, School of Sciences, European University Cyprus, 1516 Nicosia, Cyprus; g.pamboris@euc.ac.cy

**Keywords:** overhead athlete, patient-reported outcome, questionnaire, cross-cultural validation study, upper-extremity, physical function

## Abstract

Athletes engaging in overhead activities often face injury risks, emphasising the need for reliable assessment tools. This study focused on the translation and psychometric evaluation of the Kerlan-Jobe Orthopaedic Clinic (KJOC) Shoulder and Elbow Score into Greek (Gr-KJOC) for assessing upper limb function in Greek-speaking overhead athletes. The Gr-KJOC underwent meticulous translation and adaptation processes, ensuring linguistic equivalence and cultural relevance. A sample of 60 athletes participated in the psychometric evaluation, including assessments of internal consistency, test–retest reliability, construct validity, and structural validity. The Gr-KJOC demonstrated excellent internal consistency (Cronbach’s alpha = 0.95), indicating consistent measurement of the underlying construct. Test–retest reliability was excellent (ICC = 0.95), with low measurement errors. Construct validity was confirmed through correlations with the Disabilities of the Arm, Shoulder and Hand (DASH) Questionnaire. Structural validity revealed a unidimensional structure with high explained variance (75%). No floor or ceiling effects were observed, and the questionnaire proved feasible, with an average completion time of 6 min. The Gr-KJOC emerges as a reliable, valid, and feasible tool for evaluating upper limb function in Greek-speaking overhead athletes. Its psychometric properties support its utility in clinical and research contexts, contributing to the effective assessment and management of shoulder and elbow conditions in the realm of overhead sports in Greece.

## 1. Introduction

It is widely recognised that athletes who engage in overhead activities often execute quick and forceful throwing overhead movements while serving a ball. These actions typically occur in positions that push the limits of their range of motion, thereby elevating the likelihood of shoulder or other upper limb injuries [1,2,3]. Nevertheless, the increased physical demands placed at the shoulder and elbow in overhead athletes may also affect physical performance during play [4]. Despite the presence of symptomatology in this population, research has shown that they continue to train and compete, irrespective of clinical symptom severity [5]. Experts in the field assert that prevention is key in managing overhead athletes, including evaluating and monitoring upper limb health and performance [6,7]. Patient-reported outcome measures (PROMs) are highly encouraged in this clinical population for assessing functional performance [1].

Several questionnaires have been developed in the past for the assessment of upper limb function, such as the Disabilities of the Arm, Shoulder and Hand (DASH) Questionnaire, the Upper Extremity Functional Index (UEFI), the Western Ontario Rotator Cuff (WORC) Index and many more [1]. However, the frequently employed patient-reported outcome measures designed for the general population are inadequate in capturing the functional status and performance changes specific to overhead athletes [7]. Currently, there are no specific questionnaires used exclusively for the assessment of overhead athletes, apart from the Kerlan-Jobe Orthopaedic Clinic Shoulder & Elbow Score (KJOC) [8].

The KJOC has been shown to be a valid and reliable PROM that can be explicitly used in overhead athletes [8,9]. It consists of 10 questions related to upper limb function, and the score for each question ranges from 0 to 100. The final overall score is calculated from the average of the 10 items, ranging from 0 to 100. Higher scores indicate better function. The KJOC was designed by Alberta et al. [8] to assess the functional status of the upper limb of patients involved in highly demanding overhead sports. The KJOC includes questions about the self-perceived ability to perform sport-specific movements such as hitting a ball. The KJOC has been designed not only for symptomatic athletes but also for assessing the functional performance of healthy athletes [7]. Lastly, the KJOC can also be used to examine the effectiveness of any intervention in overhead athletes with shoulder or elbow pathology [7].

Selecting the KJOC is especially important in athletes as it facilitates the identification of subtle changes in shoulder and elbow function and performance [8]. Detecting such functional changes proves valuable in formulating strategies for sports training, rehabilitation and the gradual return to sports following injury [7].

The original version of the KJOC has been translated from English into several other languages, including Korean [10], Finish [7], Italian [9], Turkish [11], Norwegian [12], German [13], Spanish [14], Dutch [15] and Persian [16]. To date, no comparable PROM exists in the Greek language, although overhead sports are highly popular in Greece. Thus, the aim of this study was to translate and cross-culturally adapt the KJOC for Greek-speaking overhead athletes and examine the reliability and validity of the Greek version of the KJOC in this population.

## 2. Materials and Methods

The authors collected data between January 2023 and January 2024. Participants were recruited from multiple sports clubs in Greece using consecutive (non-probability) sampling. The authors communicated with various sports clubs in Greece to inform them about the research project and assist in the recruitment process. To be eligible to participate in the study, athletes had to meet the following criteria: (1) to be adults (>18 years); (2) to compete and train at least two times weekly in an overhead sport, such as handball, volleyball, baseball, softball, basketball, water polo, tennis or badminton; and (3) to be able to communicate, read and write in Greek natively. Athletes were excluded if they had cognitive, communicative or psychological issues. Also, athletes suffering from neurological dysfunction or cardiovascular or pulmonary dysfunctions that led to functional limitations were excluded. All athletes signed an informed consent form before participating. The study protocol was approved by the ethics committee of the University of Peloponnese.

### 2.1. Sample Size

A minimum sample of 50 participants was considered “*good*” for the assessment of the internal consistency, floor and ceiling effects, construct validity, test–retest reliability and measurement error based on the recommendation of the Consensus-based Standards for the selection of health Measurement INstruments (COSMIN) [17]. A prior analysis was also performed to establish the sample size for assessing test–retest reliability and construct validity. This analysis involved a single-group design with two measurements (test–retest), considering an effect size (δ) of 0.25, a power of 0.95 and a significance level (α) of 0.05, resulting in 54 subjects [18]. Finally, it was advised to adhere to the recommendation of maintaining a minimum subject-to-item ratio of at least 5:1 for conducting exploratory factor analysis (EFA) and Principal Component Analysis (PCA) [19]. Given that the final version of the KJOC includes 10 items intended for factor analysis, it was recommended that the minimum study sample comprise at least 50 athletes [19]. Considering potential losses to follow-up, a minimum sample of 60 athletes was deemed necessary.

### 2.2. Translation and Adaptation into Greek

The translation and cross-cultural adaptation procedures of the KJOC were based on previously reported studies [13,20,21]. The first step included the forward translation of the English version into Greek by two independent bilingual translators who were Greek in origin. One of the two forward translators was a Lecturer in physiotherapy with more than 30 years of experience in clinical physiotherapy and academic teaching. The other forward translator had no physiotherapy or medical background and was unaware of the existence of the KJOC. The two forward translators compared their translations until a consensus was reached. A single Greek version of the scale was formed from the two reports and the comments of the two translators. Subsequently, another two bilingual independent translators completed two backward translations of the Greek version of the KJOC into English. The backward translators compared the scale with the translated version to confirm whether the semantic, conceptual and experimental equivalence was met. A bilingual committee consisting of the translators reached unanimity on a satisfactory pre-final version of the KJOC in Greek, comparing all their translations with the original version.

The pre-final Greek version of the KJOC was then pilot-tested in 10 overhead athletes. Of these 10 athletes, 5 were symptomatic and 5 were asymptomatic in the shoulder region. After completion of the 10 pilot pre-final versions of the KJOC, an interview was conducted individually with all 10 athletes. Two physiotherapists with more than 10 years of experience conducted the interviews. In each individual interview, the participants were asked to explain if the content of the pre-final version of the scale was clear after reading the instructions, the items and the responses. Furthermore, they were asked to clarify whether parts of the scale were unclear and to provide suggestions for possible modifications that could have improved clarity. Feasibility was evaluated by examining the time needed to complete the questionnaire, the ease of its completion and the percentage of incomplete questionnaires (those lacking responses for more than 10% of the items).

The entire scale was found to be well conceivable by all patients, and no changes were made to the pre-final version. All participants were informed about: (a) the research objectives, (b) the absolute confidentiality of their responses and (c) their rights regarding the study. Before their involvement, each patient provided a signed consent document. Furthermore, they fulfilled a questionnaire concerning demographics and their engagement in physical and sports activities, ensuring a thorough exploration of their background and participation in the research.

The authors distributed the questionnaire in hard copy format, with two of the authors (EP and AC) overseeing the administration and providing detailed explanations of all procedures to the participants. Athletes autonomously completed the questionnaire prior to their training session at the designated training site, scheduled for 7 pm. The questionnaire completion took place at least 20 min before the commencement of the training session, and the authors were available for any necessary clarifications.

### 2.3. Reliability

Test–retest reliability was examined in the final Greek KJOC (Gr-KJOC) version. All participants (*n* = 60) were asked to complete the Gr-KJOC twice. The first time they completed the Gr-KJOC was during their first contact with the authors, and the second was 3–5 days later. The interval of test–retest sessions in this study was specified to minimise recall and be suitably short to guarantee clinical stability between testing sessions [22]. The clinical stability of the participants was examined by asking each one whether they believed that their symptoms were the same in the retest session [23]. Only patients who answered that their symptoms were the same in the retest session completed the Gr-KJOC a second time. Completion of the Gr-KJOC twice allowed the investigators to examine test–retest reliability by comparing the results of the test and retest sessions. Internal consistency was also assessed based on the degree to which separate items of the Gr-KJOC related to each other [24].

### 2.4. Validity

The construct validity was examined after correlating the results of the Gr-KJOC with the Greek Version of the Disability of the Arm, Shoulder and Hand (DASH) and the DASH Sports Module (DASH-SM) [22]. The Greek DASH has been shown to be a reliable (r′ = 0.91) and valid instrument (Correlation Coefficient = 0.62) that can provide a standardised measure of patient-centred outcomes in Greek-speaking patients with upper limb disorders, while the DASH-SM assesses symptoms and the functional status of the upper limb in sports settings [25]. The DASH contains 30 questions: 21 are related to function, 6 are related to symptom severity and 3 are related to social function. Each question is rated on a 5-point scale ((1), no difficulty; (2), mild difficulty; (3), moderate difficulty; (4), severe difficulty; (5), unable). The questionnaire score is calculated by applying established formulae for the first 30 questions, and the scores range from 0 (the best) to 100 (the worst) [25]. The DASH-SM contains 4 questions, and the goal of the DASH-SM is to identify the specific difficulties that athletes might experience but that may not affect the activities of their daily lives and, consequently, may go undetected in the 30-item portion of the DASH [22].

### 2.5. Floor and Ceiling Effect

Verification of the floor and ceiling effect was made by the percentage (>15%) of participants who obtained the minimum and maximum scores in the Gr-KJOC, respectively [17].

### 2.6. Statistical Analysis

All data was analysed using IBM SPSS statistics 29.0. Descriptive statistics were calculated and reported for all measures. The statistical level of significance was set at *p* < 0.05. The normal distribution of the data was examined through visual inspection of the Q–Q plots.

#### 2.6.1. Internal Consistency

Internal consistency, as a degree of homogeneity of the single items of the Gr-KJOC, was examined using Cronbach’s alpha. Internal consistency was considered acceptable for the Gr-KJOC if the alpha value was within the recommended range of 0.70 to 0.95 [22,26].

#### 2.6.2. Reliability (Measurement Errors)

The Intraclass Correlation Coefficient (ICC) for absolute agreement was used to examine test–retest reliability of each item and the total score of the Gr-KJOC. Measurement errors from using Gr-KJOC were estimated from the Standard Error of Measurement (SEM) and the Minimum Detectable Change (MDC). The following equation was used for the SEM: SEM = SD√(1-ICC). SD was the pooled SD calculated by the following equation: SDpooled = √(SD12 + SD22)/2. The MDC was then calculated using the following equation: MDC = SEM × 1.64 × √2, reflecting the smallest detectable within-person change in score [22,27,28]. The mean difference between the test–retest scores was graphically represented using a Bland–Altman plot, which included 95% limits of agreement. This plot showcased the relationship between the mean of the two measurements and the corresponding mean difference.

#### 2.6.3. Construct Validity

Construct validity of the Gr-KJOC was examined by correlating the results of the Gr-KJOC with the DASH and the DASH-SM. Spearman correlation was used to examine construct validity. Correlation coefficients of 0.70–0.89, 0.40–0.69 and 0.10–0.39 were considered strong, moderate and weak, respectively [29]. Structural validity was tested by Principal Component Analysis (PCA) with varimax rotation to determine the dimensionality of the overall scale [13,30]. Only factors with eigenvalues ≥ 1 were considered [30,31].

## 3. Results

The process of translation and adaptation proceeded smoothly, with no significant challenges encountered. Overall, the original questionnaire’s structure was preserved. The 10 items comprising the KJOC were successfully incorporated into the Greek version, known as the Gr-KJOC, aligning with the concerns pertinent to overhead athletes in Greek-speaking contexts. Primary translations into Greek and subsequent back translations into English revealed minor linguistic inconsistencies, which were effectively addressed during discussions within the synthesis group. Additionally, nuances of the Greek language, such as gender-specific terms for athletes and coaches, were taken into account. Consequently, we implemented gender-neutral language throughout the questionnaire. The questionnaire was completed without incident within an average duration of 6 min.

The final Gr-KJOC was completed by 60 athletes (7 male) with a mean age of 21.3 (±3.1). The rest of the participants’ demographic characteristics are shown in Table 1.

### 3.1. Internal Consistency

**Figure 1:**
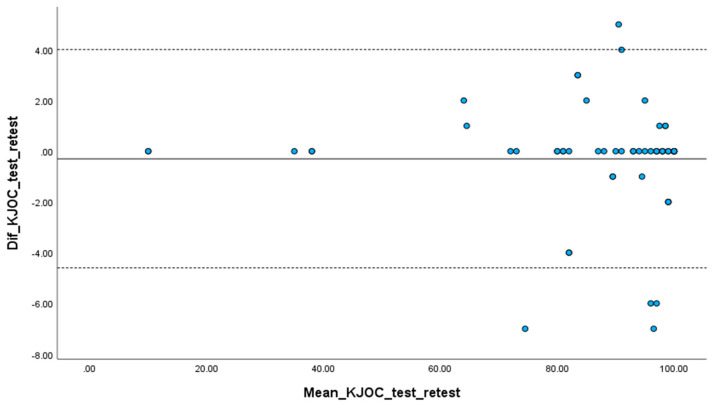
Bland–Altman plot showing the test–retest results of athletes who completed the Gr-KJOC questionnaire. *Solid line*: Mean difference between Gr-KJOC test and retest scores. *Dashed lines*: 95% limits of agreement.

Internal consistency for the Gr-KJOC was evaluated as excellent in the test (Cronbach’s a = 0.95) and retest sessions (Cronbach’s a = 0.95). Furthermore, removing any item resulted in a Cronbach’s alpha value close to the overall value (Cronbach’s Alpha if Item Deleted—Table 2). Pearson’s correlation between each item and the sum of all the other items was evaluated from the “Corrected Item-Total Correlation” of the first test session to assess whether all items measure the same underlying construct [32]. The examination did not uncover any correlation coefficients below 0.3, suggesting that all elements assessed the same underlying concept [32]. The intraclass correlation coefficient (ICC) for the Gr-KJOC was 0.95, with a 95% confidence interval of 0.94 to 0.97, indicating excellent test–retest reliability. The SEM was 6.5, and the MDC was 15.1. The findings regarding absolute reliability (SEM and MDC) were of higher level, indicating variability in the repeatability results. Test–retest reliability of each item was excellent (>0.93—Table 2). The Bland–Altman plot showed a small mean difference (Figure 1).

### 3.2. Validity

Correlating the Gr-KJOC completed in the first assessment with the DASH and the Optional Sports Module of the DASH (DASH-SM) allowed us to examine the convergent validity. The Gr-KJOC demonstrated moderate and significant correlations with the DASH (r = −0.43, *p* < 0.05) and the DASH-SM (−0.40, *p* < 0.05).

**Figure 2:**
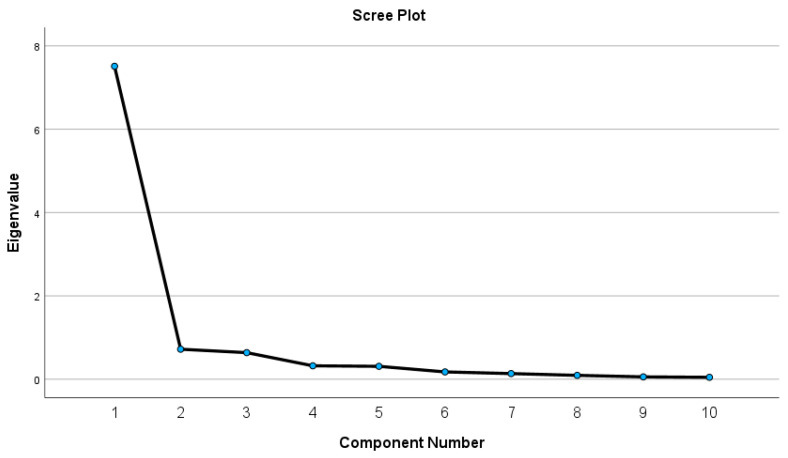
Scree plot of the principal component analysis.

Regarding the structural validity of the Gr-KJOC, principal component analysis showed one underlying factor with an explained variance of 75% and an eigenvalue of 7.5 (Figure 2). This finding further highlighted the unidimensionality of the Gr-KJOC. The Kaiser–Meyer–Olkin measure of sampling adequacy yielded a value of 0.903, indicating a relatively robust factor analysis, and individual KMO measures were all greater than 0.7, indicating the adequacy of sampling. Additionally, Bartlett’s test of Sphericity was significant, signifying that the correlations between items were substantial enough to conduct a Principal Component Analysis.

### 3.3. Floor and Ceiling Effect

Taking into account that the KJOC’s highest achievable score is 100 and the lowest is 0, we scrutinised the entire scale as a unified dimension. Our analysis of the total scale showed no indications of floor or ceiling effects in over 15% of our sample.

## 4. Discussion

The translation and cross-cultural adaptation of the Kerlan-Jobe Orthopaedic Clinic (KJOC) into Greek, referred to as the Gr-KJOC, aimed to address the absence of a comparable Patient-Reported Outcome Measure (PROM) in the Greek language for assessing upper limb health and performance in overhead athletes. The meticulous process of translation and adaptation, involving forward and backward translations and pilot testing, ensured linguistic equivalence while considering the cultural context of Greek-speaking athletes. The final Gr-KJOC maintained the original questionnaire’s structure, with minor linguistic adjustments to suit the nuances of the Greek language. The findings of this study offer evidence supporting the psychometric properties of the Gr-KJOC within a young and relatively adequate sample of Greek-speaking overhead athletes. The findings affirm that the Gr-KJOC is a dependable and valid questionnaire for evaluating shoulder and elbow conditions in Greek overhead athletes.

The psychometric properties of the Gr-KJOC were extensively evaluated. Internal consistency, assessed by Cronbach’s alpha, demonstrated excellent reliability in the initial test and retest sessions. Removing any item did not significantly alter the overall consistency, indicating that each question contributed consistently to the measurement of the underlying construct. The majority of the findings of this study are in line with the findings reported in previous studies evaluating the internal consistency of the KJOC in other languages, including Korean [10], Finish [7], Italian [9], Turkish [11], Norwegian [12] and German [13].

Test–retest reliability, assessed through the Intraclass Correlation Coefficient (ICC), showed excellent reliability for each item and the total score of the Gr-KJOC. The Standard Error of Measurement (SEM) and Minimum Detectable Change (MDC) further characterised the measurement errors, providing valuable insights into the scale’s precision. These findings are in accordance with the findings of previous studies that translated the KJOC into Korean (ICC = 0.95) [10], German (0.94) [13], Italian (0.95) [9], Turkish (0.93) [11], Norwegian (0.96) [12] and Spanish (0.96) [14]. It is important to highlight that elevated absolute reliability values, as indicated in this study, can have an impact on the repeatability of the results [7]. Similar high absolute reliability values were observed in previous research that focused on translating and assessing the reliability and validity of the KJOC in Finnish. Research suggests that older athletes may exhibit a wider range of symptoms and may observe themselves more closely, contributing to variations in the accuracy of reporting [12]. In our study, the inclusion of athletes with a relatively young mean age (21.3) could account for the observed higher absolute reliability values. Future study may consider this when recruiting athletes for conducting a similar study.

It is important to acknowledge that, in this study, none of the 10 items of Gr-KJOC was close to 1, indicating redundancy [14]. Moreover, test–retest reliability values were consistent with studies that administered the KJOC on paper [7,8,9,13,16] or online via smartphones [12]. The athletes recruited in other studies that examined the psychometric properties of the KJOC in different languages, including Korean [10], Finnish [7], Italian [9], Turkish [11], Norwegian [12] and German [13], had a mean age that ranged from 18.1 to 26.6. These studies included athletes from a variety of overhead sports, such as volleyball, baseball, softball, swimming and tennis. The participants in the studies had various previous shoulder injuries, including tendinopathies, bursitis, instabilities and fractures. Additionally, one study had an almost equal ratio of symptomatic to asymptomatic athletes [12], ensuring that their findings are equally representative in both categories. Most of the studies had more asymptomatic than symptomatic participants, as in our case [9,10,13,15]. Overall, our findings highlight that the reliability of the KJOC remains stable across different versions, diverse populations and various data collection methods.

Construct validity was examined by correlating the Gr-KJOC with established tools, the Disability of the Arm, Shoulder and Hand (DASH) and the DASH Sports Module (DASH-SM). The Gr-KJOC demonstrated moderate and significant correlations, supporting its convergent validity. Similar findings were found in similar studies that adapted the KJOC into German [13], Dutch [15], Persian [16], Norwegian [12] and Spanish [14] and reported moderate and significant correlations with the DASH. Although the DASH and DASH-SM are viewed as similar assessment tools [33], their questions might not capture the nuanced needs of high-functioning overhead athletes as effectively as the KJOC. Consequently, we anticipated lower correlations between them. Nonetheless, these tools are presently the sole Greek options for comparing fundamental aspects such as symptoms, functional ability and upper extremity performance.

Structural validity, assessed through Principal Component Analysis (PCA), revealed a unidimensional structure with a highly explained variance, affirming the Gr-KJOC’s coherence and consistency in measuring upper limb function in overhead athletes. This finding is in line with the results from the confirmatory factor analysis of the original questionnaire [8] and the principal component analysis of the Turkish and German versions of the KJOC [11,13].

No floor or ceiling effects were observed, suggesting that the Gr-KJOC adequately captures the range of upper limb conditions in the study population. The feasibility analysis showed that the questionnaire could be completed within an average of 6 min, underscoring its practical utility in an athletic setting with time constraints. Lastly, the questionnaire’s feasibility was established, and participants in the pilot study confirmed its readability and comprehension.

### 4.1. Limitations

This study has certain limitations. First, it does not offer data on the responsiveness of the Gr-KJOC, which could have provided valuable insights into the instrument’s capacity to measure changes over time and evaluate intervention outcomes [13]. Also, concerning the statistical analysis of structural validity using principal component analysis, it is worth noting that confirmatory factor analysis could have been considered as an alternative and potentially superior method of investigation. However, opting for confirmatory factor analysis might have limited the ability to compare results with previous publications [11,13]. Moreover, given that the Gr-KJOC is not tailored to any specific sport and participants in this study were recruited from various sports, the study results may only reflect the reliability and validity of the combined group, lacking specificity for individual sports. Nevertheless, the existing literature does not provide evidence that athletes from different sports demonstrate distinct psychological responses in expressing subjective functional status [34].

The re-test period was 3–5 days in order to ensure clinical stability of our sample and minimize loss to follow-up since these athletes were examined during a very intense training period with frequent competitions that could also have increased the risk of change in symptom status. Although short retest periods for reliability assessment of questionnaires may increase the risk of recall bias, a prolonged interval between administrations in a sport setting can be impractical and carries a significant risk of attrition bias [35,36]. Therefore, in the current study, we deliberately chose a brief time frame to maintain clinical stability, mitigate the risk of recall bias, and minimize the likelihood of participant drop-out. Finally, it is worth noting that we did not assess the psychometric properties of the Gr-KJOC separately, as only 13% of our sample displayed symptoms, and conducting statistical analysis on such a small sample is not recommended [17,31].

### 4.2. Practical Implications

The successful translation and adaptation of the Gr-KJOC holds practical significance for assessing upper limb health and performance in Greek-speaking overhead athletes. The meticulous translation process ensures linguistic equivalence and cultural relevance, making the Gr-KJOC suitable for this specific population [37]. Its completion efficiency, within an average of 6 min, and the absence of floor or ceiling effects make it a feasible tool for addressing the time constraints of an athletic setting,. Additionally, the Gr-KJOC’s ability to capture a comprehensive range of upper limb conditions and its correlations with established instruments underscore its practical utility in providing nuanced insights for high-functioning overhead athletes [38]. Despite certain limitations, the Gr-KJOC emerges as a valuable and culturally relevant tool for assessing shoulder and elbow conditions in this population, contributing to improved healthcare practices and research endeavours.

## 5. Conclusions

In summary, the Gr-KJOC exhibited sound psychometric properties, featuring high internal consistency, excellent test–retest reliability and moderate construct validity. The questionnaire’s unidimensional structure coupled with the absence of floor or ceiling effects underscores its suitability for assessing shoulder and elbow conditions in this particular population. Nevertheless, to establish a more comprehensive understanding of the Gr-KJOC score’s validity, additional studies involving diverse overhead sports and larger sample sizes are essential. Moreover, further investigations into the responsiveness of the score are warranted to enhance its applicability in clinical and research settings.

## Figures and Tables

**Table 1:** Participants’ characteristics.

Characteristics	Results
Age (mean, sd)	21.3 (±3.1)
Gender	7 male 53 female
Hand dominance	50 Right 3 Ambidextrous 7 Left
Years of experience (mean, sd)	10.2 (±4.2)
Previous shoulder injury (yes/no)	12/60
Competing with upper limb pain (yes/no)	8/60
Sport	47 Volleyball 11 Basketball 2 Swimming

**Table 2:** Test re-test reliability (measurement errors).

Questions	ICC	Corrected Item-Total Correlation	Cronbach’s Alpha if Item Deleted
KJOC1	0.98	0.563	0.965
KJOC2	0.95	0.737	0.959
KJOC3	0.98	0.907	0.951
KJOC4	0.98	0.902	0.951
KJOC5	0.98	0.741	0.958
KJOC6	0.97	0.835	0.954
KJOC7	0.96	0.912	0.952
KJOC8	0.98	0.848	0.954
KJOC9	0.97	0.942	0.950
KJOC10	0.93	0.882	0.953

Abbreviations: ICC: Intraclass correlation coefficient between test–retest for each question; Corrected item-total correlation: Pearson’s correlation between the specific item and the sum of all the other items; Cronbach’s alpha if item deleted: how the calculated Cronbach’s alpha value would change when each specific item is removed from the scale.

## Data Availability

Data is available upon reasonable request.
